# Effects of Noise Stress on Neuronal Activation in Rat Auditory Pathway-Related Brain Regions

**DOI:** 10.3390/diagnostics15212720

**Published:** 2025-10-27

**Authors:** Duygu Gök Yurtseven, Alper Vatansever, Gonca Topal, Şule Mergen, Ömer Faruk Özdemir, İlker Mustafa Kafa, Gökhan Göktalay, Özhan Eyigör

**Affiliations:** 1 Department of Histology and Embryology, Faculty of Medicine, Bursa Uludag University, 16059 Bursa, Türkiye; goncatopal.1992@gmail.com (G.T.); oeyigor@uludag.edu.tr (Ö.E.); 2 Department of Anatomy, Faculty of Medicine, Bursa Uludag University, 16059 Bursa, Türkiye; avatansever@uludag.edu.tr (A.V.); imkafa@uludag.edu.tr (İ.M.K.); 3 Department of Pharmacology, Faculty of Medicine, Bursa Uludag University, 16059 Bursa, Türkiye; mergensule@gmail.com (Ş.M.); ofarozdemir@gmail.com (Ö.F.Ö.); goktalay@uludag.edu.tr (G.G.); 4 Department of Biomaterials, Graduate School of Natural and Applied Sciences, Bursa Uludag University, 16059 Bursa, Türkiye

**Keywords:** anatomy, neuroscience, auditory pathway, c-Fos, rat, stress

## Abstract

**Background/Objectives:** Environmental noise is a non-specific biological stressor that is becoming an escalating health concern for both industrialized and developing countries. A study by the World Health Organization identified ambient noise as the second most prevalent factor adversely affecting public health, causing high levels of stress. Extended or intense exposure to environmental noise (EN) has been linked to various alterations in auditory pathways and auditory-related central nervous system structures. We tested the hypothesis that acute exposure to intense noise could lead to such alterations. Therefore, the aim of this study is to evaluate neuronal activation in auditory-related brain regions resulting from acute noise exposure using immunohistochemistry processes. **Methods:** We examined a total of 12 Wistar rats (6 rats for noise exposure group; 6 rats for the control group). The noise exposure group was exposed to intense noise, while the control group experienced basal noise for thirty minutes. After scarification of the rats, tissues were collected and examined histologically using the immunohistochemical staining method. **Results:** Our research demonstrates that acute noise exposure markedly elevates neuronal activity in critical parts of the auditory system, such as the cochlear nuclei, inferior colliculi, trapezoid body, and primary auditory cortex. While we identified c-Fos immunoreactive neurons in the medial geniculate body of both the experiment and control groups, no statistically significant changes were found between these groups. **Conclusions:** These findings indicate that noise exposure-related stress could be caused primarily by the disruption of lower centers rather than the medial geniculate body.

## 1. Introduction

Today, people are exposed to environmental stimuli in different ways throughout their daily routine. Changes to environmental conditions and the presence of real or perceived threats could be perceived as stress-triggering factors for both humans and animals [1]. Acute or chronic stress that is caused by these situations could lead to morphological changes in multiple systems [2,3,4,5].

Environmental noise (EN) is described as a non-specific biologic stress factor, and for both developed and developing nations, noise poses an increasing health risk [4,6,7,8,9]. According to a World Health Organization report, EN is listed as the second most common factor that negatively affects public health [10]. EN can disrupt metabolism in a similar way to other stress factors [11]. It has long been recognized that EN is harmful to auditory systems, such as cochlear hair cells, auditory nerve fiber terminals, and superior cortical structures [12].

Acute noise exposure, from short-term, high-intensity sound events caused by construction noise, sirens, concerts, and personal audio devices, is a prevalent but often overlooked source of stress. Chronic exposure, on the other hand, originates from common sounds such as traffic, industry, and home appliances. Both types of exposure are linked to hearing problems as well as mood, cognition, and heart health [13].

Although it is recommended by the Environmental Protection Agency and the World Health Organization that noise levels should not exceed 70 decibels (dBA) to protect public health [14,15], recent reports indicate difficulties in controlling and stabilizing noise pollution [16]. Furthermore, studies have also shown that many employees work in noisy workplaces [17,18,19,20]. Hearing loss caused by noise exposure is preventable; despite this, approximately 16% of people worldwide experience noise-induced hearing loss [21,22]. Besides hearing loss and auditory dysfunctions, noise exposure can cause various non-auditory health problems, such as depression, dementia, cognitive weakness, and cardiovascular disorders by, affecting vital organs [23,24,25].

Textbook knowledge indicates that the superior colliculus does not have efferent fibers for auditory pathways, but afferent fibers [26]. The superior colliculus plays a central role in processing mainly visual information, which is integrated to initiate motor commands. As such, it receives input from various centers to modify behavioral responses [27]. Nevertheless, the effects of noise stress on the superior colliculus have not been evaluated. The most decussating fibers originating from the anteroventral cochlear nucleus are known as the trapezoid body. This prominent assemblage of fibers operates by relaying auditory impulses from the cochlear nucleus to the superior olivary complex, which is essential for binaural hearing and sound source localization [28].

The audiogenic stress-responsive circuit comprises a network of brain regions that process loud or frightening sounds and coordinate appropriate behavioral and physiological stress responses. The cochlea initially detects sound impulses and transmits them to auditory-related brain regions for perceptual processing and the amygdala to evaluate their emotional value [29,30,31]. The amygdala subsequently activates the hypothalamus and the periaqueductal gray (PAG) to trigger defensive reactions and engage the hypothalamic–pituitary–adrenal (HPA) axis, leading to the secretion of stress hormones [32,33]. This integrated circuit facilitates the swift identification of detrimental sounds and orchestrates the adaptive reactions vital for survival.

Defects in the brain stem response mechanism, one of the most critical steps of hearing pathways, can cause many disorders, including hearing loss, tinnitus, mental disorders, and synaptopathy [34,35,36,37]. A recent study indicated that long-term exposure to community noise is linked to 36% higher odds of mild cognitive impairment and a 29% increase in the risk of developing Alzheimer’s disease [38]. The cochlear nucleus, located in the brain stem, is critical for controlling and regulating auditory pathways [39]. Recent studies have used different methods to evaluate changes in the cochlear nucleus for different noise-induced disorders, such as tinnitus and hearing loss [40,41].

The Fos, a proto-oncogene’s protein product, has frequently been utilized to demonstrate variations in neuronal activity linked to environmental stressors [42,43]. Campeu and Watson (1997) reported evidence of noise-induced c-Fos mRNA expression in various cortical and subcortical centers, especially the auditory cortex [44]. Current studies are focusing on the effects of chronic acoustic stress on different cortical centers by evaluating c-Fos activity [45,46,47]. Moreover, increased c-Fos expression was found in the cortical center in relation to auditory pathways and other cortical centers in the tinnitus model [48]. Neuronal activity may be assessed using transcription factors like c-Fos, which is the protein encoded by the proto-oncogene. Quantifying c-Fos expression in neurons under physiological settings can reveal the active system [49].

Previous studies have mainly evaluated the effects of chronic noise exposure on the auditory cortex, its connections with the limbic system, and the impacts that excessive amounts of noise have on auditory disorders. Despite substantial developments, we still do not fully understand how c-Fos activation varies in different auditory nuclei, especially in acute noise scenarios. However, the impact of intense noise exposure on the superior colliculus and its functions in noise-induced stress pathogenesis have yet to be assessed. Therefore, there is limited knowledge about the function the superior colliculus has in noise-induced stress. We hypothesized that subcortical regions beyond auditory pathways, such as the superior colliculus, could lead to intense noise-induced stress. Based on gaps in the current literature, this study aimed to investigate how neurons in the cochlear nuclei, inferior colliculi, superior colliculi, MGB, medial nucleus of the trapezoid body, and auditory cortex are activated by the exposure of rats to acute dynamic white noise.

## 2. Materials and Methods

### 2.1. Experimental Animals

All animal experiments were conducted in accordance with the National Institute of Health Guide for the Care and Use of Laboratory Animals and approved by the University’s Animal Care and Use Committee (Degree No: 2022–10/05). Adult male Wistar rats (*n* = 12; 3–4 months old; 300–400 g weight) were obtained from the author’s institution at the Experimental Animals Breeding and Research Center two weeks prior to experimental procedures. We considered that female rats experience estrous cycles, leading to variations in their physiological and behavioral responses. Such fluctuations can introduce confounding variables in behavior-based studies, such as anxiety, stress response, and pain sensitivity [50]. The number of animals in each group was determined according to previous studies [51,52,53]. The rats were housed in pairs in a quiet, temperature and humidity-controlled room (maintained 20 ± 2 °C and 55 ± 5% humidity) under a 12 h:12 h light–dark cycle (lights were on between 07:00 a.m. and 07:00 p.m.). Food and water were provided ad libitum. Handling and habituation of the rats began after one week of acclimatization. To minimize environmental and housing effects, the control and experimental rats were transferred to the testing room for 4 h daily for one week prior to the experimental stimuli. The rats were randomly allocated into two groups: control (*n* = 6) and noise exposure for 30 min (*n* = 6).

In this experiment, animals were randomly assigned to either the control or noise exposure group. c-Fos immunohistochemical staining and image analysis were carried out by an investigator blinded to the experimental groups to prevent observer bias.

Preyer’s reflex test was performed on all animals to assess auditory function and animals showing negative or weak responses were excluded from the study.

### 2.2. Noise Exposure

**Figure 1:**
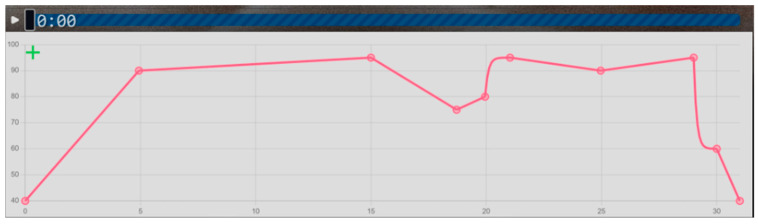
Noise exposure profile across a 31 min session. The sound intensity (dB—*y* axis) was modulated over time (*x* axis) according to a predefined pattern. Key data points included 0.00 min—40 dB; 4.93 min—90 dB; 15.00 min—95 dB; 18.72 min—75 dB; 19.95 min—80 dB; 21.03 min—95 dB; 24.97 min—90 dB; 29.00 min—95 dB; 30.00 min—60 dB; and 31.00 min—40 dB.

A custom-developed protocol was required to generate a precise, complex, and dynamic acoustic profile (as detailed in Figure 1), which is not achievable with standard commercial noise exposure systems. Continuous white noise (50 Hz–16 kHz; 40 dBA–105 dBA) was generated using an online tool (https://mynoise.net/NoiseMachines/whiteNoiseGenerator.php, accessed on 14 March 2023) and amplified via an Ibanez IBZ 10 G amplifier (output: 10 watts, 8 Ω, and 6.5-inch speaker). Because direct control over volume transitions was limited by the website’s default setting, sound levels were programmatically adjusted using a custom-developed plugin (https://cdn.jsdelivr.net/gh/tenazur/deneme@main/voltime1.3.js, accessed on 14 March 2023).

Prior to experiments, the entire system was calibrated. A calibration curve was generated by mapping the programmatic volume settings of the JavaScript plugin (in 5% increments) to the resulting dBA level measured by the sound-level meter (RadioShack^®^ 33-2055 set to ‘A’ for weighting and ‘slow’ for response). The microphone was placed in the center of the test cage at the approximate height of the animal’s head. This process confirmed that the script’s commands accurately produced the acoustic profile shown in Figure 1. Sound levels were confirmed daily before the first session.

The amplifier was positioned 150 cm away from the test cage. Noise levels were calibrated using RadioShack^®^ Digital Sound Level Meter, RadioShack Corporation, Fort Worth, TX, USA (Model: 33-2055) at the animals’ exposure site. Animals from each group were individually transferred to the test cage for 30 min sessions; the control group experienced only ambient noise (60 dBA), whereas the noise exposure group was subjected to white noise ranging between 40 and 105 dBA according to a predefined acoustic profile designed to minimize habituation (Figure 1) [46]. This profile included linear and nonlinear (logarithmic) transitions, structured as follows:

- Initial Increase (0–5 m): The sound level increases linearly from 40 dB to 90 dB, representing a rapid onset of auditory stimulus.
- Slight Rise (5–15 m): A slower, continued linear increase occurs, reaching a peak at 105 dB.
- Drop in Intensity (15–18 m): The noise level then decreases linearly to 75 dB, representing a partial release from high-intensity noise.
- Logarithmic Rise (18–20 m): A nonlinear, more gradual logarithmic increase brings the level back to 95 dB.
- Fluctuations (20–30 m): The profile exhibits mild oscillations between 90 and 95 dB, simulating fluctuating environmental noise levels.
- Final Drop (30–31 m): A rapid drop occurs, with decibel levels falling sharply from around 90 dB to the baseline of 40 dB, marking the termination of the auditory stimulus.

The noise exposure room was acoustically isolated to prevent environmental sounds. Animals were not anesthetized and were individually placed in a 50 cm × 50 cm × 50 cm cage to prevent them from covering their ears against the cage walls, with external observation conducted.

### 2.3. Tissue Preparation

After completing the 30 min exposure period, rats were transferred to another acoustically isolated room and allowed to rest for 90 min to facilitate c-Fos protein expression [49]. Subsequently, animals were deeply anesthetized with intraperitoneal injection of xylazine (10 mg/kg) + ketamine (70 mg/kg) followed by transcardiac perfusion fixation with 4% paraformaldehyde in phosphate buffer (pH 7.4, 300 mL per animal). Brains and brainstems were carefully dissected, post-fixed overnight, and sectioned coronally at 40 µm thickness using a Leica VT1000S vibratome, Leica Microsystems GmbH, Wetzlar, Germany. Sections were transferred into a Tris-HCl buffer (0.05 M, pH 7.6) and stored at −20 °C in cryoprotectant solution until immunohistochemical staining was performed.

### 2.4. c-Fos Immunohistochemistry

The tris-HCl buffer was utilized for all washing steps. The sections were incubated in blocking buffer (10% normal horse serum, 0.2% Triton X-100, and 0.1% sodium azide in Tris-HCl buffer) for 2 h to minimize non-specific binding. The sections were subsequently incubated in rabbit anti-c-Fos antibody (1:10,000 dilution; Chemicon, Billerica, MA, USA), followed by donkey anti-rabbit IgG secondary antibody (1:300 dilution; Jackson ImmunoResearch Laboratories, West Grove, PA, USA) for 2 h. Visualization involved processing sections using an avidin–biotin complex kit (ABC Elite Standard Kit, Vector Laboratories, Burlingame, CA, USA) for 1 h and staining with diaminobenzidine (DAB) solution (25 mg DAB, 2 g nickel ammonium sulfate, 2.5 µL hydrogen peroxide in 100 mL Tris-HCl buffer). The sections were washed, mounted onto slides, dried, and cover-slipped using a DPX mounting medium.

Quality control assessments included an inspection of both auditory and non-auditory brain areas for c-Fos-positive neuronal labeling.

### 2.5. Evaluated Brain Areas Cell Counting

**Figure 2:**
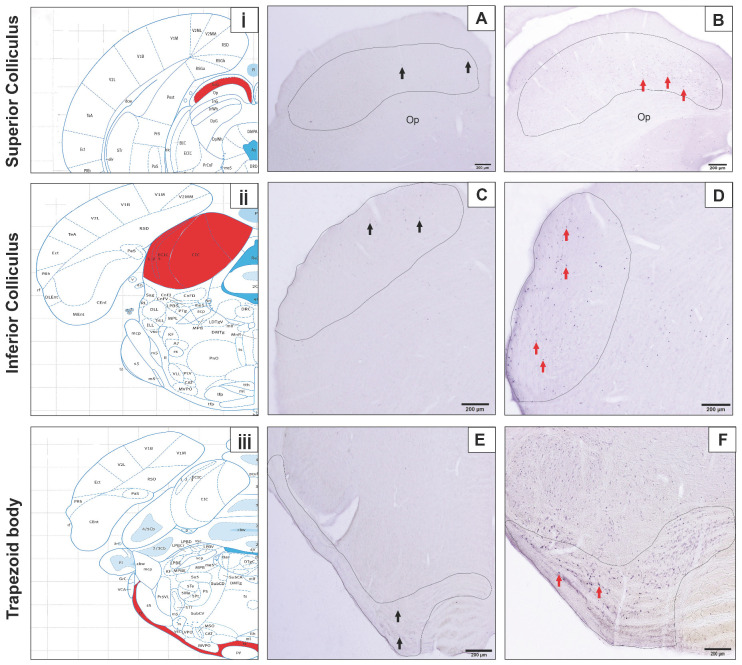
(**i**,**ii**,**iii**) Localization of the superior colliculus (SuG), inferior colliculus (ECIC and CIC), and medial nucleus of the trapezoid bodies (tz) is marked with red areas according to Paxinos and Watson’s rat atlas (2007) [54]. (**A**,**C**,**E**) Micrographs are taken from control rats, (**B**,**D**,**F**), micrographs are from experimental groups, and the arrow points to c-Fos-positive neurons. We used 10× magnification; Op: optic nerve.

**Figure 3:**
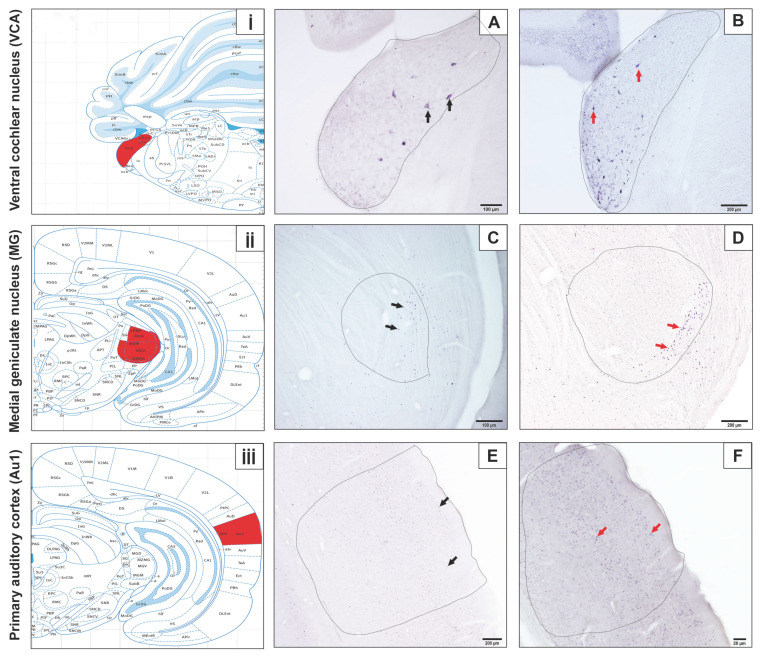
(**i**,**ii**,**iii**) Localization of the ventral cochlear nucleus (VCA), medial geniculate body (MGB), and primary auditory cortex (Au1) is marked with red areas according to Paxinos and Watson’s rat atlas (2007) [54]. (**A**,**C**,**E**) Micrographs are taken from the control, and (**B**,**D**,**F**) and micrographs are from the experimental groups. Red and black arrows point to c-Fos-positive neurons. We used 4× and 10× magnification.

Immunostained sections were analyzed using an Olympus BX–50 photomicroscope (Tokyo, Japan) equipped with a digital camera (Olympus DP71 CCD color camera, 1.5 million pixels). Evaluated brain regions included the ventral cochlear nucleus (VCA) (bregma −9.12 mm to −10.56 mm), MGB (bregma −4.80 mm to −6.48 mm), primary auditory cortex (Au1) (bregma −3.24 mm to −6.84 mm), trapezoid body (bregma −8.88 mm to −11.04 mm), superior colliculus (bregma −5.28 mm to −7.80 mm), and inferior colliculus, subdivided into the external cortex (ECIC; bregma −7.20 mm to −9.24 mm), central nucleus (CIC; bregma −7.80 mm to −9.24 mm), and dorsal cortex (DCIC; bregma −8.16 mm to −9.24 mm) (Figure 2 and Figure 3) [54]. Counting was performed on sections using consistent rostrocaudal coordinates for each animal in ImageJ software, version 1.54e.

### 2.6. Statistical Analysis

The mean ± standard errors of the data were determined in each group, with IBM SPSS 20 (Armonk, NY, USA) used for analysis. We compared differences in the brain areas between the control and experiment groups using the Mann–Whitney U test. Statistical significance was defined as *p* < 0.05.

## 3. Results

c-Fos immunoreactivity was identified in the neuronal nucleus through dark blue-black staining. Microscopic analyses revealed that c-Fos-positive neurons were localized within the auditory cortex, cochlear group, MGB, and superior and inferior colliculi (Figure 2 and Figure 3). In evaluated brain regions, neurons displaying blue-black immunopositivity for the c-Fos protein were classified as activated.

Table 1 summarizes the total values of cell counts, mean values, minimum and maximum counts, standard deviations, and associated *p*-values.

Increased c-Fos expressions were found in neurons within the auditory cortex, cochlear nuclei, superior and inferior colliculi, and medial nucleus of the trapezoid body in the experiment groups compared to the control groups (*p* < 0.01). However, the MGB showed no statistically significant differences between the experiment and control groups (*p* = 0.68). These results emphasize the critical involvement of specific auditory pathways in response to experimental conditions and underscore the importance of these brain regions in auditory processing.

**Figure 4:**
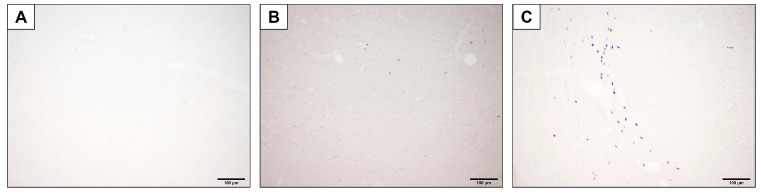
Representative images of the medial geniculate nucleus showing negative control staining (**A**), c-Fos immunoreactivity in the control group (**B**), and c-Fos immunoreactivity in the experimental group (**C**).

The specificity of immunohistochemical staining was assessed through control studies. Control staining was performed simultaneously with the main immunohistochemical staining, omitting the primary antibody in step 4. No staining was observed during control staining. This result accurately verified the specific labeling of the antibody (Figure 4).

## 4. Discussion

The present findings demonstrate that acute noise triggers significant neuronal activation (indexed by c-Fos expression) in multiple levels of the rat central auditory pathway, including the cochlear nuclei, trapezoid body, inferior colliculi, and primary auditory cortex; however, the MGB is unaffected.

Transcription factors such as c-Fos (the protein product of the c-Fos gene) are used as markers to determine changes in neuronal activity [49]. Although c-Fos expression in neurons is minimal in basal conditions, it increases under certain physiological conditions; by determining this increase, information can be obtained for the activated system. The immunoreactivity of the c-Fos protein, which is synthesized in an experimentally stimulated neuron, translocated to the nucleus, and involved in genetic activation in the neuron, can be determined within 60–90 min following the stimulus [48]. The immunohistochemistry technique used in this study is one of the primary techniques used in functional anatomical mapping of neuroendocrine systems; localizations of the c-Fos protein in neuronal nuclei were also determined [54,55].

Noise exposure adversely impacts both peripheral and central auditory regions. Exposure to loud noise can result in lasting alterations to hearing thresholds, leading to the dysfunction of auditory cells [56,57]. Groschel, M. et al. reported that the effects of noise volumes higher than individual thresholds can cause damage to neurons within hours and last for an extended duration, particularly in the lower auditory pathway [58]. These consequences worsen with repeated exposure to noise; however, some buildings can mitigate further harm. Individuals with normal hearing exposure to noise have diminished otoacoustic emissions and reduced efficacy of inhibitory processes in the efferent auditory system. This indicates that damage exists despite hearing thresholds remaining normal [57,58].

Noise exposure has a significant effect on the MGB, which could lead to cell death and changes in cell function. Chronic auditory stress has been shown to cause apoptosis in the MGB, specifically in its ventral, medial, and dorsal parts. This leads to a higher death rate among cells from 24 h to 14 days after exposure [59,60]. Noise exposure lowers metabolic activity in the MGB and other parts of the auditory pathways. However, stimulating the MGB with high-frequency sounds may increase neuronal activity, which could help treat conditions like tinnitus [61,62]. Long-term exposure to moderate noise during essential stages of development has been linked to a lower density of neurons in the MGB. This shows how sensitive this structure is to sound stress [63]. These results emphasize the importance of the MGB for processing sound and how easily loud noises can damage it. However, according to our results, while the number of c-Fos positive neurons was higher in the experiment group than the control group, statistical results did not identify any significance. Nevertheless, increased c-Fos activity in both groups suggests that the primary cause of acoustic stress may stem from a disorder prior to MGB damage.

Additionally, Campeau and Watson (1997) [44] demonstrated that rats exposed to noise levels between 90 and 105 dB elicited robust, intensity-dependent increases in c-Fos mRNA levels across several auditory brainstem structures, including the cochlear nuclei, superior olivary complex (encompassing the trapezoid body fibers), nuclei of the lateral lemniscus, and inferior colliculus. Notably, they reported heightened c-Fos expression within the medial division of the MGB at the highest sound intensities [44]. These findings suggest that specific subregions of the auditory thalamus are engaged in the acute noise stress response. In contrast, the present study did not detect significant differences in overall c-Fos protein expression in the MGB between the noise-exposed and control groups. This discrepancy may arise from methodological variations, such as assessing the c-Fos protein versus mRNA, or from anatomical differences in region-specific analysis (assessing the entire MGB vs. its subdivisions). The medial division projects to limbic regions and may be the only MGB subnuclei to activate c-Fos. By contrast, he ventral MGB, the main lemniscal relay, may already be near maximal activity under baseline conditions (e.g., 60 dB ambient noise in controls). Similarly, Campeau et al. (1997) observed that MGB injuries eliminated noise-induced c-Fos expression in downstream limbic and hypothalamic areas without affecting lower auditory nuclei [64]. Despite its lack of acute c-Fos activation, the MGB is crucial in transmitting audiogenic stress signals to stress-regulatory regions in the forebrain. Our work found evidence of strong c-Fos induction in the auditory cortex of noise-exposed rats, showing efficient acoustic stress signal transmission without an increase in MGB c-Fos expression. Acute 30 min noise exposure may have activated a pre-existing pool of thalamocortical neurons, increasing cortical c-Fos without MGB gene transcription. Rapid habituation or thalamic inhibitory gating may restrict c-Fos expression. Our observation that acute noise exposure did not significantly elevate c-Fos expression in the MGB suggests that limbic activation may have occurred due to subcollicular projections or through specific MGB subdivisions, such as the medial division, which were not independently analyzed. Regardless, it is evident that acute noise robustly engages both the auditory brainstem and cortical regions, which likely interact with stress-responsive centers to generate the physiological and behavioral outcomes associated with noise exposure.

Burow et al. undertook a similar experimental design to our study protocol. However, while these researchers evaluated mRNA levels of the c-Fos oncogene protein, we assessed c-Fos protein expression. While the exact correlations between c-Fos expression and transcription, or between c-Fos expression and depolarization in a specific neuron, were weakly correlated, immunohistochemistry with c-Fos antibodies serves as a potent instrument for examining dynamic neuroanatomy [49,65]. The authors reported increased mRNA, rather than protein levels, in similar brain regions to the present study. Although their results revealed increased c-Fos mRNA levels in all auditory-related brain regions, our results indicate that c-Fos protein expression is not statistically different between the control and noise groups. Based on this finding, it may be more useful to evaluate protein expression rather than mRNA to evaluate the neuronal activation of c-Fos [66]. The time at which sampling is conducted following stimulus must be carefully considered. These results show that the audiogenic circuit coordinates both behavioral and neuroendocrine stress responses. Disruption of this circuitry, such as sustained inferior colliculus hyperactivity or protracted amygdala activation, may increase susceptibility to seizure or anxiety-like behaviors [67,68,69,70]. Thus, knowing the circuit’s functional dynamics, particularly its plasticity and activation thresholds, is essential for determining the various impacts of audiogenic stress on different experimental paradigms.

Our findings further support the notion that the cochlear nucleus and inferior colliculus, as key early auditory centers, are particularly sensitive to acute noise stress. These lower-level structures exhibited the highest c-Fos elevations, suggesting that they serve as primary sites for noise-induced neuronal excitation. This observation aligns with their known roles in encoding sound intensity and mediating reflexive responses, such as the acoustic startle reflex. Similar patterns have been observed in humans: an fMRI study in young adults with normal hearing revealed that broadband noise elicited widespread activation across the central auditory pathway, including the cochlear nucleus, superior olivary complex, lateral lemniscus, inferior colliculus, MGB, and auditory cortex [71]. Notably, individuals with higher noise exposure over their lifetime demonstrated greater fMRI responses to sound onset within these regions compared to those with lower levels of noise exposure, despite having normal audiometric thresholds [71]. This suggests that repeated noise exposure may lead to central hyperactivity or plastic alterations in auditory processing networks. Consistent with these findings, our results revealed strong c-Fos induction in both brainstem and cortical auditory neurons following acute noise exposure. Together, animal and human data suggest that intense sound triggers a cascading activation pattern extending from the cochlear nuclei to the auditory cortex. The absence of a significant increase in c-Fos expression in the rat MGB observed in our study may point to the subthalamic and cortical circuits as primary nodes for propagating acute acoustic stress. Nevertheless, it is important to emphasize that the integrity of the MGB remains essential for initiating downstream neuroendocrine stress responses to auditory stimuli [64]. Thus, acute noise exposure can activate the auditory brainstem and cortex, structures likely responsible for generating perceptual and reflexive outputs, while the MGB operates as a key conduit to higher-order and limbic centers, even if its immediate early gene response is comparatively muted.

The superior colliculus (SC) has no direct function when transmitting auditory information through the auditory pathways [72]. However, it is instrumental in modulating stress responses, especially in reaction to noise and various sensory inputs. It synthesizes multisensory information and plays a key role in protective behaviors, such as active avoidance, which is essential during stressful events [73,74,75]. The SC exhibits heightened neuronal activity in response to salient stimuli, enabling prompt responses such as escape when confronted with potential threats, including auditory cues linked to danger [73,76]. The SC is an essential subcortical region that significantly regulates autonomic responses, including heart rate and changes in vascular function, particularly during heightened stress. This complex interaction highlights the profound link between sensory processing and bodily responses. The SC functions as a crucial integration hub where diverse sensory inputs converge, allowing the body and mind to adapt to stresses, such as abrupt loud sounds or unforeseen visual stimuli [77]. In light of previous studies, the SC is activated not only as a result of prolonged noise exposure but also acute stress related to exposure to loud noises. Similar to previous studies, our results demonstrate that activation of the SC increased under acute noise exposure. However, it could be hard to explain whether the SC directly functions in auditory pathways, since its activation has been demonstrated in various stress situations.

Acute noise exposure reveals distinct patterns of neuronal activation. The 30 min stimulus used in the present study induced a rapid surge in c-Fos expression across auditory and stress-related brain regions, providing a “snapshot” of neuronal populations activated by a novel stimulus. Our study likely captured the peak of this initial activation phase, as evidenced by the strong c-Fos induction in auditory circuits following acute noise exposure. The same regions might exhibit diminished c-Fos responses with repeated exposure or re-exposure, consistent with the well-established phenomenon of stress habituation. Repeated stimulation typically leads to the attenuation of both HPA axis activation and immediate-early gene expression [44]. Interestingly, the orbitofrontal cortex has been implicated in modulating this habituation process. c-Fos mRNA studies have demonstrated sustained activity in this region during chronic noise exposure, suggesting its role in downregulating the stress response [64]. Therefore, our current results typically reflect the neural activation profile characteristic of the acute phase and its expected shifts with prolonged or repeated exposure to the same acoustic stressor [64].

## 5. Conclusions

In conclusion, acute noise exposure elicits widespread neuronal activation across the auditory pathways, consistent with our expectations. However, contrary to prior findings, the MGB did not exhibit a statistically significant increase in c-Fos expression between the control and experimental groups. This discrepancy suggests that the stress effects of acute noise may originate from disruptions occurring at lower auditory levels, before the MGB. Our findings highlight the importance of further investigating subcortical auditory regions, such as the olivary nuclear complex, as potential targets for intervention. Furthermore, the correlations between auditory pathways and limbic system structures are crucial for understanding the molecular mechanisms of acute noise-related stress responses. Understanding how specific nuclei within the auditory system contribute to stress-related activation patterns could facilitate the development of targeted treatments to mitigate the impact of acute acoustic trauma. Moreover, since c-Fos and related markers map changes in neuronal activation, they play an invaluable role in charting the neuroanatomy of noise stress and guiding strategies for auditory rehabilitation and stress reduction.

## 6. Limitations

A key limitation of the present study is the absence of behavioral and endocrine assessments, which restricts the interpretation of functional consequences associated with observed neuroanatomical changes. To comprehensively understand the impact of acute noise stress on the central auditory system, future research should integrate broader molecular, behavioral, and hormonal analyses. Additionally, employing variable noise intensities and durations could help clarify how different acoustic parameters modulate stress-related responses across auditory- and stress-related brain regions.

## Figures and Tables

**Table 1:** Neuron counts and statistical results.

	Control Group	Experiment Group	
Brain Area	Mean Value (With SD, Min and Max Numbers)	Mean Value (With SD, Min and Max Numbers)	*p*-Value
Auditory Cortex	441.5 ± 176.08 (171 to 664)	1113.13 ± 475.25 (584 to 1839)	<0.001
Medial Geniculate Body	308.83 ± 122.75 (191 to 471)	373.33 ± 155.91 (199 to 654)	0.68
Superior Colliculus	508.16 ± 186.23 (214 to 703)	1515.16 ± 743.09 (684 to 2822)	<0.001
Inferior Colliculus	233.83 ± 115.39 (78 to 380)	1478.33 ± 824.1 (689 to 2905)	<0.001
Medial Nucleus of the Trapezoid Body	43.16 ± 37.43 (2 to 93)	772.16 ± 373.05 (255 to 1167)	<0.001
Cochlear Nucleus	345.33 ± 192.33 (84 to 633)	706.5 ± 172.32 (557 to 1022)	<0.001

## Data Availability

The original contributions presented in this study are included in the article. Further inquiries can be directed to the corresponding author.

## Abbreviations

The following abbreviations are used in this manuscript:

MGB	Medial geniculate body
SC	Superior colliculus
VCA	Ventral cochlear nucleus
Au1	Primary auditory cortex
TZ	Trapezoid body
